# Video consultation for non-acute headache by neurologist is not inferior to traditional consultations in terms of headache consultation rate at follow-up: *post hoc* analyses of a randomized controlled trial

**DOI:** 10.3389/fneur.2026.1798381

**Published:** 2026-04-07

**Authors:** Svein I. Bekkelund, Kai I. Müller

**Affiliations:** 1 Department of Clinical Medicine, UiT-The Arctic University of Norway, Tromsø, Norway; 2 Department of Neurology and Clinical Neurophysiology, University Hospital of North Norway, Tromsø, Norway; 3 Department of Neurology, Hospital of Southern Norway, Kristiansand, Norway; 4 Institute of Clinical Medicine, University of Oslo, Oslo, Norway

**Keywords:** e-consultation, headache, randomized controlled trial, RCT, return consultation, telemedicine

## Abstract

**Objective:**

To investigate whether use of video consultations at neurologist for patients with new non-acute headache are noninferior to face-to-face consultations in the need for subsequent headache consultations or hospitalizations.

**Methods:**

This study is based on *post hoc* analyses from data collected at baseline consultations (*n =* 402) and by questionnaires at 12-month follow-up (*n* = 291) in a randomized controlled non-inferiority efficacy and safety trial. The study is conducted in a combined rural and urban area where neurological service is centralized to one hospital. Rate of patients consulting neurologist for headache or being hospitalized for headache during 12-month follow-up were compared between groups consulted by video and face-to-face. Secondarily, follow-up headache visits in general practice were investigated by questionnaire.

**Results:**

Rates of patients in the video group (*n* = 22; 16.8%) and the face-to-face group (*n* = 24; 18.5%) who consulted neurologist or were hospitalized for headache the next 12 months after baseline consultations were statistically indifferent, CI [0.637, 2.273], (*p* = 0.752). Median (IQR range) number of consultations or hospitalizations were 0 (IQR: 0–5) respectively 0 (IQR: 0–6), *p* = 0.421. Rates of patients visiting general practitioners (GPs) for headache during 12-month follow-up were 89 (63.1%) respectively 92 (69.2%), *p* = 0.309. Median (IQR range) were 1.0 (0–15) and 1.0 (0–15), *p* = 0.156. Sex, age, waiting time to specialist, renewed headache diagnosis and initiation of medical treatment by specialist were equally distributed between the groups. No secondary headache or underlying medical conditions were detected in any group.

**Conclusion:**

Acknowledging the methodological limitation of the *post hoc* design, these data give evidence to the view that use of video in headache treatment at specialist contribute to better health service efficiency by providing availability to care for these patients.

**Clinical trial registration:**

ClinicalTrial.gov, identifier NCT02270177.

## 1 Introduction

Headaches are common complaints in the population and a frequent reason for people to seek healthcare (1). Migraine is estimated as the second most burdensome neurological disorder after stroke and Alzheimer’s disease (2, 3). Reports over time show that headache syndromes are underdiagnosed and undertreated (4, 5). Variable access to headache specialists across countries emphasizes the need for more knowledge about alternative and easier consultation forms like use of video or other electronic devices in clinical practice (6, 7). Additionally, a shared organization of consultation practice between specialist services and general practitioners available to the patients for optimal headache care is not always provided and needs to be better coordinated (8, 9). Thus, telehealth research for headache has grown (10, 11). Patients with neurological disorders including headache and other pain conditions are well treated with telemedicine (12–15). Additionally, patients with difficult headache may benefit from video consultations (15, 16). Also from a specialist point of view, headache and follow-up consultations were reported to be well suited for video consultation (17). Likewise, a multicenter RCT study found diagnostic accuracy to be favorable in 212 migraine patients via an online system (18). Neither treatment outcome, safety, sufficiency of technology, nor patient satisfaction via video were inferior to traditional consultation for headache patients visiting a neurological specialist (10, 15, 19–21). Headache patients reporting positive experience with tele consultations in a resent Italian study, related their future priority of the method to time saving (22). Lack of randomized studies investigating how video consultations at neurologist affect subsequent headache consultation activity motivated the present study. Furthermore, to ensure access to headache care during the covid-19 pandemic period, use of digital consultations has become more relevant (21, 23).

The purpose of the study was to test the hypothesis that video consultation for new non-acute headache patients referred to an outpatient neurological clinic from general practitioners (GPs) is not inferior to traditional face-to-face consultations in terms of subsequent headache follow-up consultations/hospitalizations at specialist (primary endpoint) and at GPs (secondary endpoint).

## 2 Materials and methods

### 2.1 Study design and recruitment

**Figure 1:**
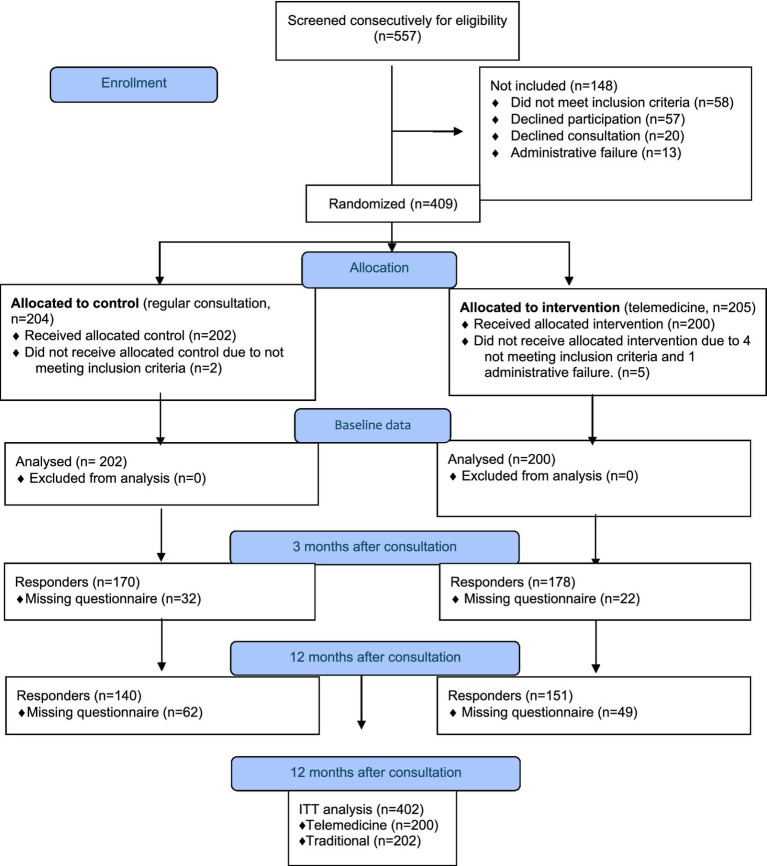
Flow diagram of patients referred to neurologist from general practitioners for headache and randomized to video consultation or face-to-face consultation.

The present study is a prospective, single-center, open label, randomized, controlled noninferiority clinical trial (ClinicalTrials.gov; id. NCT02270177). Headache patients (*n* = 402) consecutively referred from GPs to the neurological outpatient clinic at the Department of Neurology, University Hospital of Tromsø. Norway from 30th September 2012 until 30th March 2015 were identified, screened, randomized to either video- or face-to-face consultations, and then consulted by a neurologist (Figure 1). The hospital serves more than 265.000 inhabitants over a 48.618 km^2^ land area, and almost ¾ live in rural areas. No alternative public or private neurological services exists in this area. The present *post hoc* study was not originally included in the protocol.

### 2.2 Randomization and sample

Inclusion criteria were: (1) Patients referred from GPs to neurologist for headache, (2) no evidence of secondary headache, i.e., headaches classified as primary headache without specific causes (24) except patients with suspected medication overuse headache, (3) Norwegian speaking men and women aged 16–65 years, (4) no previous consultation by neurologist for headache during the past 2 years, (5) waiting time from referral to consultation ≤4 months. Non-inclusion criteria were opposite of inclusion criteria and inability to give free informed consent.

### 2.3 Consultation standards and inferiority margin

A study nurse welcomed the patients at entrance of the Neurological Department, the Tromsø University Hospital, controlled the self-administered prefilled forms and consents, and called upon the randomization administrator (Centre for Quality Improvement and Development at the hospital). After being block-randomized, they were guided to the examination room for face-to-face consultations or to the videoconference room. Video consultations were performed by using a video conference system: Cisco C40 Integrator package, Multisite and Touch-Control Device for C Series; Cisco, San Jose, California, USA, Cisco Precision HD 1080p 12x camera. Further details are published elsewhere (20). Video consultations and face-to-face consultations were performed at the same place. The study nurse confirmed that the web-camera and microphone worked well and were operated correctly by the patients. A short training to assure optimal communication with the specialist was provided before the consultations started. A standardized interview developed before the trial was used during the consultations along with check lists for inclusion criteria and diagnostic classification as published by the International Headache Society. The non-inferiority margin was defined by <15% impairment in outcome measures in the video-group compared to the face-to-face group (20). A 15% difference in clinical measurement is usually regarded as acceptable because it generally falls within the range of biological or methodological variation or, in other words, being part of the allowable total bias of the study. to achieve valid results beyond variations in biology, endpoint measurement and statistical analyses.

### 2.4 Data collection and endpoint assessment

We obtained demographic, social, clinical and endpoint data by self-administered prefilled forms, structured interview at baseline and by questionnaire at one-year follow-up. The prefilled forms included Headache Impact Test-6 (HIT-6) measuring 6 headache impact items (pain, social-, role- and cognitive functioning, vitality, and psychological distress). Every question was answered by “Never, Rarely, Sometimes, Very Often or Always,” and each answer scored, respectively, 6, 8, 10, 11, or 13 points (25). Pain intensity score using a horizontal visual analogue scale (VAS) ranging from 0 to 10 (0 = no pain, 10 = worst possible pain) was prefilled by the patients (26). We used the International Classification of Headache Disorders-2 (ICHD-2) to diagnose the patients (24). The same neurologists conducted both video and face-to-face consultations (KIM and SIB). A complete overview of the selected variables is published elsewhere (20). At 12-months follow-up, a questionnaire addressing demographics, clinical and headache characteristics and endpoint variables was sent to the patients either through an online survey service (Questback) or by a postal letter. Non-responders received a reminder 2 weeks later.

Endpoints: Neurological specialist consultations and hospitalizations for headache during follow-up (primary endpoint) were recorded via the Hospital’s patient documentation system. “Have you consulted your GP for headache after the specialist consultation?” (“Yes” or “No”) and “number of headache consultations at GP after the specialist consultation” (secondary endpoint) were obtained from questionnaire. Medication: painkillers, triptans, and preventive headache drugs used in the last month, chronic neck pain; continuously presence of neck pain during the last three months, insomnia (DSM-IV criteria) were some of the recorded clinical characteristics.

### 2.5 Statistical analysis

Descriptive variables are presented as mean and standard deviation (SD) or median and interquartile range (IQR) in variables with skewed distribution as observed for number of neurological and GP consultations during follow-up. Consequently, comparisons of endpoints defined by continuous data between the two randomized groups were performed by two-sided independent Student *t*-test, respectively, Mann Whitney U-test. Categorical variables were presented as numbers and % while data used for group comparisons analyzed by Chi-Square Test and confidence intervals. Level of statistical significance was set at *p* < 0.05. Data were analyzed with SPSS (version 29, IBM Corp).

## 3 Results

### 3.1 Descriptive analyses

One-year response-rates after neurological specialist consultation were 151/200 (75.5%) in the video-group and 140/202 (69.3%) in the face-to-face group, *p* = 0.119 (Table 1). Mean values and group comparisons of patient’s characteristics at baseline and one-year follow-up can be found in Table 1. A majority had migraine as primary headache and there were not found statistically differences in any dimension being compared between the two groups (Table 2). Medical treatment was initiated in about 4/5 of the patients in any group, most commonly Triptans (Table 2). Neither were there any differences in drug prescriptions, and the patients were generally satisfied with the consultation at specialist (Table 2). Waiting time to specialist (about 2 months), and rates of patients receiving new diagnosis and/or drug treatment were all similar between the groups (Table 3). However, consultation time was about 5 min shorter in the video group (Table 3). By reviewing the hospital’s patient’s records, we did not detect any case of dangerous headache or an underlying medical condition that could be associated with headache.

**Table 1:** Clinical characteristics in randomized groups of 402 Norwegian patients referred to specialist for headache consulted by video or face-to-face.

	Baseline			12-month follow-up		
	Video (*n* = 200)	Face-to-face (*n* = 202)	*p*-value	Video (*n* = 151)	Face-to-face (*n* = 140)	*p*-value
One-year response (%)				151/200 (75.5)	140/202 (69.3)	0.119
Females (%)	148 (74.0)	153 (75.7)	0.774	119 (78.8)	103 (73.6)	0.362
Age, years, mean (SD)	36.0 (13.0)	38.0 (13.7)	0.124	36.7 (13.2)	39.3 (14.2)	0.096
Education, years, mean (SD)	13.5 (3.0)	13.8 (3.1)	0.222	13.5 (2.9)	13.8 (3.1)	0.516
Sick leave due to headache, *n* (%)	58 (29.0)	62 (30.7)	0.791	42 (27.8)	40 (28.6)	1.0
BMI (kg/m^2^)	27.1 (5.4)	26.9 (5.3)	0.617	27.1 (5.4)	27.1 (5.1)	0.505
Obesity, BMI ≥ 30, *n* (%)	52 (26.0)	49 (24.3)	0.731	42 (27.8)	39 (27.9)	1.0
Chronic neck pain, *n* (%)	89 (44.5)	99 (49.0)	0.370	67 (44.4)	64 (45.7)	0.906
Insomnia, *n* (%)	61 (30.5)	65 (32.2)	0.748	41 (27.2)	42 (30.0)	0.676
Hypertension, *n* (%)	14 (7.0)	22 (1.9)	0.221	11 (7.3)	18 (12.9)	0.172

Baseline and 12-month follow-up data of 291 patients are presented with mean (SD) or numbers (%). BMI, body mass index; SD, standard deviation.

**Table 2:** Headache characteristics and treatment in randomized groups of 402 Norwegian patients referred to specialist for headache consulted by video or face-to-face.

	Baseline			12-month follow-up		
	Video (*n* = 200)	Face-to-face (*n* = 202)	*p*-value	Video (*n* = 151)	Face-to-face (*n* = 140)	*p*-value
Onset of headache, age, mean (SD)	24.5 (14.4)	25.4 (14.3)	0.533	24.4 (14.6)	26.6 (15.2)	0.203
Headache duration, years, mean (SD)	13.9 (13.0)	12.4 (12.6)	0.436	13.9 (12.7)	13.5 (13.5)	0.711
HIT-6, mean (SD)	64.1 (6.1)	64.0 (6.1)	0.824	63.7 (6.3)	63.7 (6.1)	0.988
VAS, mean (SD)	7.1 (2.2)	6.9 (2.1)	0.492	7.0 (2.2)	6.9 (2.0)	0.716
Migraine, *n* (%)^a^	106 (53.0)	113 (55.9)	0.617	79 (52.3)	77 (55.0)	0.724
Tension-type headache^a^	15 (7.5)	8 (4.0)	0.188	12 (7.9)	5 (3.6)	0.177
Medication overuse headache (MOH)^a^	35 (17.5)	38 (18.8)	0.826	27 (17.9)	26 (18.6)	1.0
Drugs prescribed						
Triptans, *n* (%)	81 (40.5)	75 (37.1)	0.539	63 (41.7)	49 (35.0)	0.276
NSAIDs, *n* (%)	48 (24.0)	61 (3.2)	0.179	36 (23.8)	41 (29.3)	0.352
Antihypertensive, *n* (%)	33 (16.5)	33 (16.3)	1.0	27 (17.9)	20 (14.3)	0.429
Antiepileptic, *n* (%)	28 (14.0)	23 (11.4)	0.457	21 (13.9)	16 (11.4)	0.599
Antidepressant, *n* (%)	44 (22.0)	56 (27.7)	0.205	29 (19.2)	39 (27.9)	0.096
Patients satisfied with consultation	164 (82.0)	150 (74.3)	0.200	132 (87.4)	123 (87.9)	1.0

Baseline (*n* = 402) and 12-month follow-up data of 291 patients are presented with mean (SD) or numbers (%). BMI, body mass index; HIT-6, Headache Impact Test-6; NSAID, Non-Steroidal Anti-Inflammatory Drugs; VAS, visual analogue scale.

^a^Most prominent headache subtype given by specialist; SD, standard deviation.

**Table 3:** Waiting time to specialist, consultation time and clinical management of headache patients by neurologist and randomized to either video or face-to-face consultations.

	Baseline			12-month follow-up		
	Video (*n* = 200)	Face-to-face (*n* = 202)	*p*-value	Video (*n* = 151)	Face-to-face (*n* = 140)	*p*-value
Waiting time to specialist, days, mean (SD)	63.6 (29.5)	60.6 (26.5)	0.295	63.4 (29.5)	59.4 (26.8)	0.234
Consultation time, total, minutes, mean (SD)	38.8 (9.5)	43.7 (12.3)	< 0.001	39.4 (9.6)	43.6 (11.2)	< 0.001
Diagnostic change by specialist, *n* (%)	43 (21.5)	40 (19.8)	0.713	30 (19.9)	27 (19.3)	1.0
Additional headache diagnosis given, *n* (%)	109 (54.5)	118 (58.4)	0.481	76 (50.3)	80 (57.1)	0.290
Drug treatment initiated, total, *n* (%)	164 (82.0)	166 (82.2)	1.0	123 (81.5)	113 (80.7)	0.882
Patients satisfied with consultation, *n* (%)	158 (79.0)	156 (77.2)	0.278	134 (88.7)	127 (90.7)	0.719

Baseline and 12-month follow-up data are presented with mean (SD) or numbers (%). SD, standard deviation.

### 3.2 Endpoint analyses

Neurological and GP consultations (numbers/frequencies of patients and median numbers of consultations) within a 1-year follow-up period after baseline consultations are shown in Table 4. Frequency of combined neurological headache consultations and hospitalizations were 16.8% (video group) and 18.5% (face-to-face group) (Table 4), *p* = 0.752. This difference is within the non-inferiority margin (<15%). Also, number and rate of GP re-consultations were similar (Table 4). More than 60% of the consultations at neurologist resulted in additional headache visits in primary care at follow-up regardless of consultation form at baseline (Table 4).

**Table 4:** One-year follow-up visits and hospitalizations by neurologist in 291 patients originally randomized to either video- or face-to-face consultations for headache.

	Video (*n* = 151)	Face-to-face (*n* = 140)	Confidence intervals (CI)	*p*-value
Follow-up headache consultations by neurologist, *n* (%)	19/142 (13.4)	22/131 (16.8)	[0.674, 2.527]	0.540
Follow-up headache consultations by neurologist, median (IQR)	0 (0–5)	0 (0–6)	NA	0.420
Follow-up hospitalizations due to headache, *n* (%)	3/139 (2.2)	2/130 (1.5)	NA	NA
Follow-up neurological headache hospitalizations, median (IQR)	0 (0–1)	0 (0–2)	NA	NA
Follow-up neurological headache consultations + hospitalizations, *n* (%)	22/139 (16.8)	24/130 (18.5)	[0.637, 2.273]	0.752
Follow-up neurological headache consultations + hospitalizations, median (IQR)	0 (0–5)	0 (0–6)	NA	0.421
Follow-up headache consultations by GP, *n* (%)	89/141 (63.1)	92/133 (69.2)	[0.793, 2.166]	0.309
Follow-up headache consultations by GP, median (IQR)	1.0 (0–15)	1.0 (0–15)	NA	0.156

Mean (SD), median (IQR) or numbers (%) are presented. GP, general practitioner; IQR, interquartile range; NA, not applicable; SD, standard deviation.

## 4 Discussion

By comparing two groups of headache patients with similar social and clinical characteristics, the current study showed that use of video was not inferior to face-to-face consultations in terms of Neurological and GP headache re-consultation rates for 0–12 months follow-up. These findings may facilitate better access to specialist for these patients. and a potential for improved health service.

### 4.1 Comparisons with earlier studies

No study has systematically investigated rate of return consultation at specialist after initial consultation for non-acute headache. Non-randomized studies show that patients with neurological conditions are satisfied with follow-up consultations by telemedicine. About 90% of American neurological outpatients with chronic diseases including headache were satisfied with community-based tele-neurological follow-up visits (12). There are however, only a few previous RCT studies that compare follow-up consultation practice by GPs after being consulted by a neurological specialist, but none in relation to headache. A previous video-based RCT-study was performed among new neurological patients either treated at two small Irish hospitals without neurologist or at a neurological center. A secondary analysis of the data showed that 86 patients consulted by video did not have significantly more often neurological follow-up consultations than 82 patients consulted face-to-face (29% vs. 22%) (14). Although this study included a heterogenic group of neurologic patients with different outcome measures, the finding conforms with the present study. However, video was regarded as less efficient than face-to-face consultations due to higher rates of clinical investigations performed in the video group, but this was not studied here (14). A retrospective analysis from the same study showed that neurological patients randomized to GP consultation had more often follow-up visits than those treated by neurologist (27). It is worth noting that the consultation time was about 5 min shorter in the video group despite similar clinical features and outcomes between the groups indicating this method to be efficient.

Video consultations for headache patients at specialist yield high patient satisfaction and clinical efficiency regardless of whether they live in urban or rural areas. Also, video consultations is a good alternative to physical visits according to caregivers in both adult (28) and pediatric (29) special practices probably due to time savings. Video is less time-consuming and cheaper for the patients and the health organizers (10). Treating rural headache patient at specialist in North Norway costed almost four times more than those living in urban areas (20). Friedman reported higher convenience and shorter consultation time in a group of 22 video-treated patients with difficult migraine compared to 23 patients treated by face-to-face in a RCT study (15). In a subgroup of patients with remission from chronic headache, patients in the face-to-face group more often consulted GP for headache during the 12-month follow-up period compared to those in the video group (30). Headache sufferers are in general less satisfied with health services (8). Reasons for that are incompletely understood, but misdiagnosis, undertreatment and variable access to headache specialists are reported findings to consider (4, 6, 31). Variable use of diagnostic guidelines, especially in general practice, may affect treatment outcome and follow-up consultation practice (9). Thus, use of structured interview based on diagnostic guidelines, might have contributed to similar outcomes in the two groups here (32, 33). Living in rural areas being less supported by specialist healthcare, video consultations may reduce availability barriers (34). Referral variation related to patient and GP, and availability of the service may alter outcomes in such studies but are sparsely studied and therefore largely unexplained. Hence, headache patients referred to specialist, consulted GP more often and were more concerned about their headache than the non-referred without necessarily having more serious headaches (35). In one RCT study, women with headache living in rural areas, were less often referred to specialist despite higher pain score (20). Furthermore, patients´ negative expectations about headache consequences may influence upon the time course of the disease (36, 37). A study in 10 European countries graded the best headache care as followed: treatment at specialist > treatment by GP > self-medication (8). Hence, the diversity of factors that influence upon headache service in the society call for more RCT studies Another report from the present trial demonstrated that serious headache incidents in a populations of patients referred to specialist were rare regardless of consultation form indicating that video consultation for non-acute headache is safe (38).

### 4.2 Strengths and limitations

Although the *post hoc* design represents lower evidence level, the power seems sufficient (<15% variation). The GP-related outcome may be less valid since the GP consultations possibly represent a more heterogenic outcomes (patient seeking behavior, GP practice patterns etc). Use of questionnaire for assessment of secondary endpoint may suffer from recall bias. Data from follow-up consultations at specialist (primary endpoint) are controlled for since all neurological activity in the region is centralized to one hospital. A certain problem is lack of dropout analysis for both specialist consultation treated groups. This may reflect a certain attrition bias. Limited clinical follow-up equivalence, and lack of interim analyses are other critical aspects of. Contrary, the RCT design and consecutively inclusion of patients from general practice are features that strengthen internal, respectively, external validity of the study. In-hospital consultations may provide similar group conditions but makes the study less comparable to clinical practice, however. Newer communication platforms that probably reduce technical barriers more than older video equipment used in this study may potentially strengthen the relevance of the present findings.

## 5 Conclusion

Use of video consultations for headache by specialist may improve headache management quality in the population by giving the patients better access to the healthcare provider and stimulate to better organization of the healthcare system. The video method also seems to be safe. More studies preferably with RCT design aiming to identify patient groups suitable for video consultations both at specialist and GP and how a shared praxis at follow-up should be organized are needed. More knowledge should be gained about clinical, social and economic effects of video consultation in urban areas compared to rural areas.

## Data Availability

The raw data supporting the conclusions of this article will be made available by the authors, without undue reservation.
